# Creativity and mental health: A profile of writers and musicians

**DOI:** 10.4103/0019-5545.31516

**Published:** 2007

**Authors:** K. S. Pavitra, C. R. Chandrashekar, Partha Choudhury

**Affiliations:** Sridhar Nursing Home, Shimoga - 577204, India; * Department of Psychiatry, NIMHANS, Bangalore, India

**Keywords:** Creativity, mental health, writers, musicians

## Abstract

Creativity and its link with mental health have always been much speculated about. However there have been a handful of methodologically sound studies to clearly establish the relationship between creativity and mental health. The objective of the study therefore was to examine the psychiatric morbidity stress profile, coping skills and personality profile in creative versus non-creative populations. Forty writers, 40 musicians and 40 controls chosen after randomization, who met the inclusion and exclusion criteria constituted the sample of the study. All the subjects were administered GHQ-28; SCAN for all GHQ positives (and 10% of GHQ-ves), Perceived stress scale and coping check list and NEO-FFI. Statistical analysis was done using SPSS 11.0 version. Pearson's correlation, Chi-square and ANOVA one-way tests were used. The present study corroborated the findings of earlier studies in 70's and 80's that there was no difference between creative and non-creative groups in terms of mental illness and stress profile. The writers differed significantly from the other two groups on religious and faith domain of coping skills. The two creative groups had similar personality characteristics and scored significantly high on all dimensions compared to the non-creative group.

Creative imagination, creative motives and creative products are unique to human beings and are the source of their cultural achievement. Creativity is an ability to make new combinations and it is one of the most highly valued of human qualities. Creativity may prove to be the key to success or failure in human beings' quest for knowledge, in their journey beyond the bounds of the sure and seen and in exploration of the unknown. A creative thinker is always trying to create something new and this involves a great amount of unconscious rearrangement of symbols. In general there is a great recognition in today's living across the globe to be creative in one's everyday activity.

Though creativity has been of interest over the years; there has been no universal acceptance of its definition or of methods for its measurement. There are many definitions of creativity. However the characteristics of creative thinking given by Goertzels[1] appear relevant here.

- The product has novelty and value either for the thinker or the culture.
- The thinking is unconventional.
- It is highly motivated and persistent or of great intensity and
- The problem was initially vague and undefined so that part of the task was to formulate the problem itself.

There have been 3 distinct stages in the conceptualization of creativity. From prehistory until well into the medival period, it was generally considered to be a mysterious, supernatural process – a gift from the gods or from God, depending on the religion or the culture (Greek, Hindu, Egyptian, Incan V/s Moslem, Jewish or Christian). As the Renaissance led to humanism, the concept of inherited genius took over. Gradually psychological and contextual influences received more recognition

## Creative Individual

Since ancient times, the observation has been made that extremely creative individuals were unusual in many ways and it has been suggested that psychological processes akin to those observed in madness might be an important component of the special abilities of genius. In the research into the connection between creativity and psychopathology a theoretical connection has been drawn between creative functioning and unusual or regressed thinking processes,[2] affective symptoms,[3,4] personality traits and values[2,4] and behavioural characteristics. Almost any extraordinary performance or creative achievement, then – whether it is in writing, music, poetry, philosophy, dance, art, sculpture or intellectual discovery – could be said to be the variants of the belief that there can be “no great genius without some touch of madness”. References to famous, emotionally disturbed artists, writers, poets, composers, scientists and philosophers – Vincent Van Gogh, Franz Kafka, Edward Munch, Ezra Pound, Delmore Schwartz, William Cowper, Ernest Hemingway, Friedrich Nietzsche, Eugene O'Neill, Charles Darwin, Fyodor Dostoievsky, Robert Lowell, Sylvia Plath and others are cited widely in the literature. Likewise, results of various studies and anecdotal reports suggest an increased rate of schizophrenia, manic-depressive disorder, depression, personality disorder or alcoholism in creative individuals. While it is quite clear that emotional instability is usually detrimental to creativity, it also may be advantageous. It may provide the intense motivation, the conviction, egocentrism, the unconventionality, the imagination and the inspiration so necessary for new discoveries and breakthroughs. It may also allow the artist, writer, poet, composer and scientist to escape the powerful social and cultural constraints that mostly favor conformity and convention. What remains to be determined is just what types of psychopathology inhibit or facilitate what types of creative activity.

Whether one needs to “be sick to be creative”? Can one lead a peaceful and pleasant life and still have hopes of being innovative? Or does not have to experience abnormal extremes of some sort?

A basic difficulty is that despite the intriguing nature of such reports, the relationship between creativity and insanity may be apocryphal. Many other artists, writers, poets, composers and scientists presumably have led reasonably “sane”, emotionally stable lives – William Shakespeare, Albert Einstein, Jules Henri Poincare, Wealt Whitman, William James, Aldous Huxley, Carl Jung, Camille Pissarro, Niels Bohr, Duke Ellington and the like. Reports, such as the Stanford 35 – year follow-up study of over 1,000 “geniuses”, Ellis' psycho biographical study of eminent men, the MacKinnon study of creativity in architects and others, suggest a connection between creativity and mental health rather than mental illness.

These conflicting results leave many key questions unanswered. Is mental illness essential or merely incidental to the creative process? Do psychoses, mood disturbances, intoxications or severe characterlogical defects serve as sources of inspiration, allowing innovators to perceive reality in novel ways or do they inhibit creativity? Do mental symptoms or emotional distress represent the consequence of creative activity – the price to be extracted for relentlessly pursuing the unknown as Carl Jung might claim – or the impetus for discovery and innovation? Are many artists and writers apt to rely on alcohol and drugs to still their overactive minds or to fuel their imaginations when they are feeling emotionally blocked and intellectually inhibited? Even more to the point, are certain types of individuals, with certain kinds of psychopathology, in combination with certain other talents and abilities and under certain circumstances more likely than other types of individuals to make scientific breakthroughs or important works of art?

While a great deal of attention has been paid to dysfunctional creative individual the healthy creative person is barely represented in the literature. It might prove worthwhile to look closely at the lives of creative person with less dramatic life history since they may serve as positive models others may emulate.

Art provides a “path to the sacred and spiritual, even in a profane and fragmented world”. We need to encourage creativity and creative people in our midst. We also need to find more effective ways, to prevent, treat and destigmatise mental illnesses of all kinds. We need both good art and good science. Science and art, creativity and mental illness may all be trivialized, however and we may do more harm than good to those “afflicted” with either creativity or mental illness if we promote romantic association that over-look best evidence.

Summing up, biographical studies offer impressive indications that eminent writers as a group suffer more problems than do other eminent creators or the general population. Despite the robustness of the statistics, they must be considered cautiously. Diagnoses have often been made posthumously using loose and inconsistent diagnostic criteria. In many cases the validity of a diagnosis of mental illness, alcoholism or suicide cannot be verified. In addition, solid statistical evidence of the extent of mental illness, alcoholism and suicide in the general population is still far from precise. Finally, it is possible that the creative persons with dramatic lives and early deaths are more likely to become eminent and have biographies written about them.

Hence this study was conceived in a manner to overcome earlier methodological flaws by having a randomized sample, a control group and studying different dimensions like psychopathology, personality profile, stress profile and coping skills using structured instruments and strict inclusion & exclusion criteria.

## Aims and objectives

1. To study the prevalence of psychiatric morbidity in Creative population compared to a non-creative population.
2. To study the stress level and coping skills in Creative population Compared to a l non-creative population.
3. To study the personality profile in Creative population. compared to general non-creative population.

## Hypothesis

1. Prevalence of psychiatric morbidity is not higher in creative people (musicians and writers) compared to non-creative people.
2. Coping skills are better in creative population compared to non-creative people.
3. There is no difference in the personality profile of creative people compared to non-creative people.
4. There is no difference in the level of perceived in creative people compared to noncreative people.

## MATERIALS AND METHODS

### Sample

#### Experimental group

Forty writers and 40 musicians were recruited through randomization using lists in alphabetical order available in Karnataka Sangeeth Academy, Karnataka Sahitya Academy and Kannada and culture department. The lists consisted of 300 accomplished musicians and writers. Random numbered tables were used to choose the subjects with the help of the statistitian. The contact details were noted from the sources.

#### Control group

Forty subjects were chosen after ensuring exclusion criteria in the general population by the following method. One subject was chosen from every fourth house of forty experimental subjects after randomization. No matching was done.

#### Inclusion criteria

1. Age range of 25 to 65 years
2. Either sex.
3. Active creative work in the last 5 years in the respective fields namely literature and music.
4. Bangalore based writers and musicians with inclusion in the respective directories.
5. Availability of a written informed consent.

#### Exclusion criteria for cases

- Current acute medical or neurological illness.
- Mental retardation
- Nonavailability of written informed consent.

#### Additional Exclusion criteria for controls

- Major regular creative activity (music/ dance/ painting/ theatre) as either a hobby or profession.

#### Instruments/tools

After randomization, the subjects were screened using a checklist for the inclusion and exclusion criteria. The following instruments were used for the assessment after obtaining written informed consent (Appendix I).

- Sociodemographic data sheet (Developed for the current study).
- General Health Questionnaire (GHQ-28)[5]
- SCAN 2.1 version (WHO1998.
- Perceived Stress Scale – PSS.[6]
- NEO-5 factor Inventory[7]
- Coping check list[8]

#### Mode of recruitment

After being randomly chosen, experimental subjects were contacted through mails and telephone. The Researcher introduced herself and the purpose of the study was presented and an overview was given. All subjects who consented over the phone or replied back were interviewed. At the time of interview written informed consent was taken. The investigator interviewed each subject personally over two sessions. Randomly chosen experimental subjects were asked to introduce the researcher to the neighbourer in the fourth house from his or her house.

#### Statistical analysis

Data was expressed using descriptive statistics such as mean standard deviation for continuous variables and number and percentage for categorical variables.

The reverse coding of items was done to calculate the domain totals. Domain scores were calculated. Categorization of NEO 5 domains based on standardized scores was done.

Comparison between various groups for NEO 5 domains, coping skills domains and other continuous variables was carried out by ANOVA followed by post hoc tests. Categorical variables were analyzed using chi-square test *P* < 0.05 was considered

#### Statistically significant

All the statistical analysis was carried out by SPSS version 11.0 software.

## RESULTS

### Sample characteristics

A total of one hundred and twenty Bangalore based people, forty each in the groups of musicians, writers and noncreative who met the inclusion criteria were included in the study. There were 63 women and 57 men with a mean age of 48.4 (SD ± 11.3) years. All the subjects belonged to above middle class with a monthly income of 12,366 (± 7,570) rupees [Table 1].

**Table 1:** Sample characteristics

Age and sex distribution					
Variables	Controls	Musicians	Writers	F/χ^2^ value	*P* value
Age (mean ± SD)					
years	44.7 ± 11.68	47.6 ± 11.28	52.85 ± 9.42	5.75	0.004#*
Sex-male	20 (50%)	18 (45%)	19 (47.5%)	0.201	0.905
Female	20 (50%)	22 (55%)	21 (52.5%)		

*P*<0.05

# Controls v/s writers,

* Musicians v/s writers,

$ Controls v/s musicians.

**Table 2:** Socio-demographic characteristics

Variables	Controls	Musicians	Writers	χ^2^ value	*P* value
Education					
Metric	7 (17.5)	4 (10)	1 (2.5)		
PUC	3 (7.5)	3 (7.5)	1 (2.5)		
Graduation	25 (62.5)	19 (47.5)	5 (12.5)	42.12	0.001
Post graduation	5 (12.5)	14 (35)	33 (82.5)		
Family type					
Nuclear	22 (55)	19 (47.5)	28 (70)		
Extended	8 (20)	20 (50)	12 (30)	23.98	0.007
Joint	10 (25)	1 (2.5)	0		
Marital status					
Single	2 (5)	10 (25)	0		
Married	38 (95)	29 (72.5)	39 (97.5)	1.46	0.227
Separated	0	1 (2.5)	1 (2.5)		
Source of income#					
Creative	—	32 (80)	22 (55)	46.87	0.001
Non creative	40 (100)	8 (20)	18 (45)		
Birth place					
Urban	28 (70)	34 (85)	22 (55)	8.57	0.014
Rural	12 (30)	6 (15)	18 (45)		

# Creative people who pursued their field as the sole source of income. There were also people who were accomplished musicians/writers who had different jobs along with creative work. Figures in parentheses are in percentage.

### Socio-demographic profile

Socio-demographic characteristics of subjects in each group were comparable in domains of sex, income and experience in years. However they differed significantly on ANOVA (*P*=0.004) in terms of age as writers were older compared to the noncreative and musician groups. The duration of stay in Bangalore was greater for the musicians compared to the groups (*P*=0.001). There was significant difference in the birthplace and early childhood stay in the three groups, as musician and noncreative groups were from an urban background, the writers were predominantly from the rural background (*P*=0.014). Comparing the educational status, it was found that the writers were more often postgraduates where as others two cohorts were graduates or lower educational qualification (*P*<0.001). Musicians (25%) were more often single as compared to writers (0%) and noncreative (5%) population but this did not reach statistical significance.

### Family characteristics

Comparison of the familial characteristics was done and it was found that musicians and writers had high percentages (100 and 82.5) family history of creativity; that is either they had accomplished musicians/writers in the family or there was a family member who had at least 5 years of experience in music, writing, painting, dance or sculpture and differed significantly from the noncreative group (15%) (*P*>0.001) (Table 3A). Most of the children of the creative population pursued creative work in the form of writing, theater, music, dance and painting which was statistically significant (*P*>0.001). However it was observed that creative population had higher occurrence of mental illness in the family (*P*=0.001) and more commonly it was affective illness (*P*=0.007) and less commonly psychosis and substance abuse.

**Table 3a:** Familial characteristics

Variables	Controls	Musicians	Writers	F/χ^2^ value	*P* value
Family H/O mental illness	9 (22.5%)	23 (57.5%)	24 (60%)	14.13	0.001
Family H/O creativity	6 (15%)	40 (100%)	33 (82.5%)	71.65	0.001

**Table 3b:** Pattern of mental illness in the families

Variables	Controls(%)	Musicians(%)	Writers(%)
Bipolar disorder		1 (2.5)	
Depression	8 (20.0)	12 (30)	14(35)
Acute psychosis / schizophrenia	1 (2.5)	5 (12.5)	5 (12.5)
Suicide	1 (2.5)	3 (7.5)	4(10)
Substance use	1 (2.5)	1 (2.5)	2(5)
Mental retardation		3 (7.5%)	1 (2.5%)

**Figure 1:**
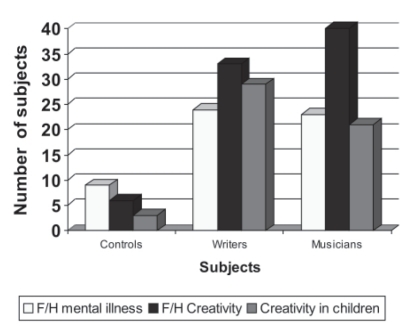
Family history

**Figure 2a:**
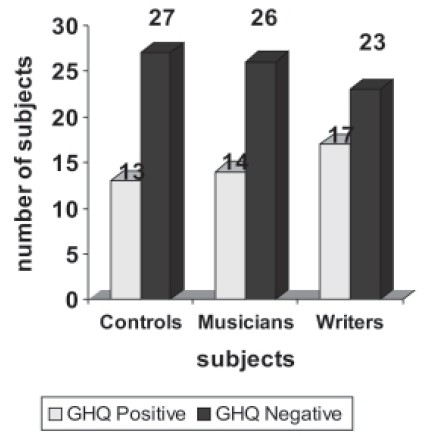
Comparison of GHQ positivity

**Figure 2b:**
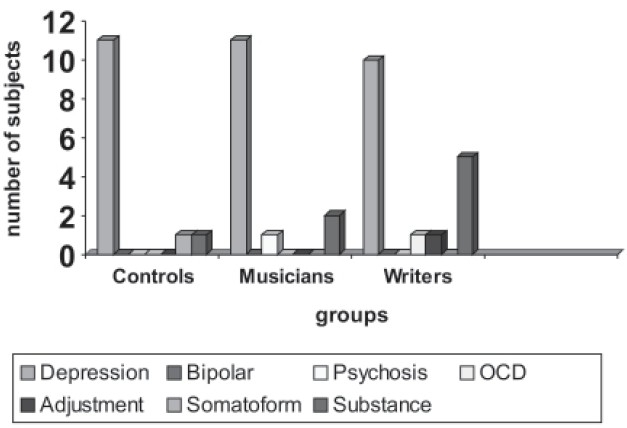
SCAN dianoses

**Figure 3a:**
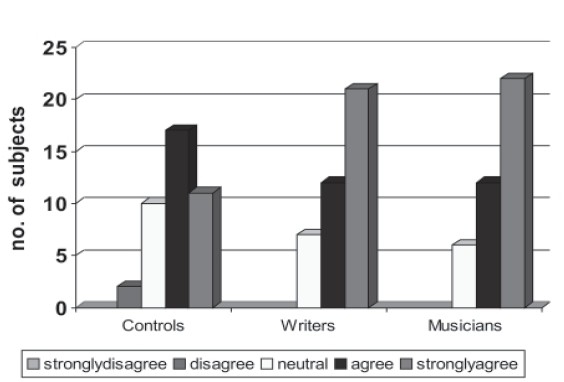
Neurotic dimension

**Figure 3b:**
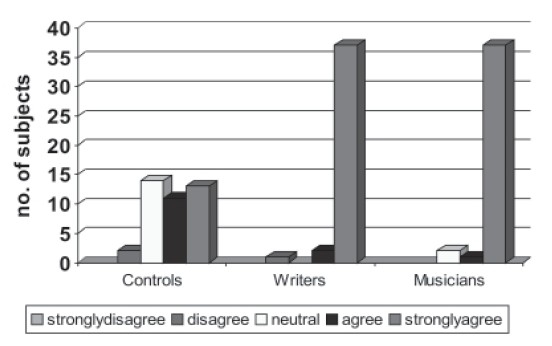
Extroversion dimension

**Figure 3c:**
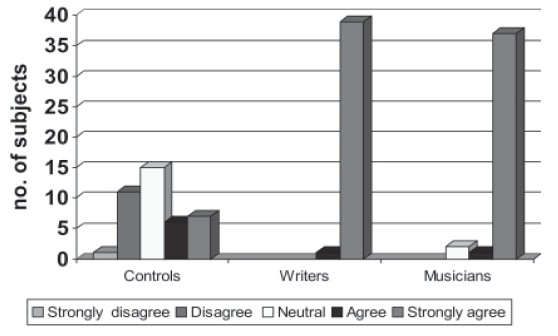
Openness dimension

**Figure 3d:**
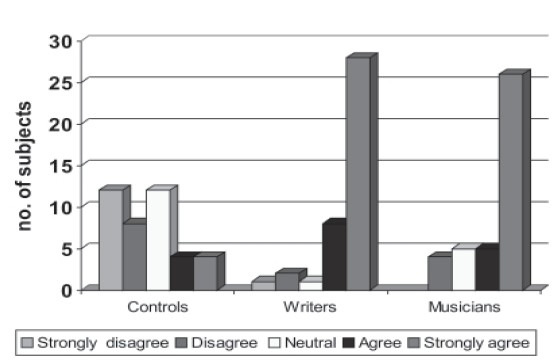
Agreebleness dimension

**Figure 3e:**
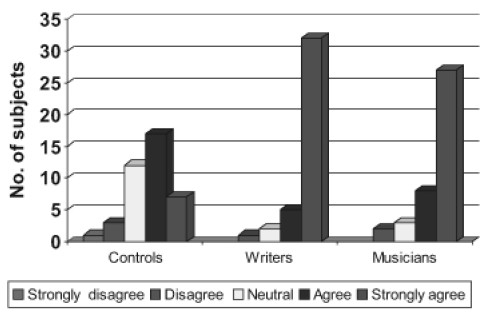
Consclentiousness dimension

### Psychopathology

All the groups were administered GHQ-28 and 44 members scored above 5 on the scale, which was the cut off to administer, SCAN. The above 44 subjects along with other 10% (8 subjects) of GHQ-28 negative population were administered SCAN 2.1 Version. Of the 52 people who were administered SCAN 2.1 Version 44 had syndromal mental illness, which was higher in the creative population though not of statistical significance. None of the 8 GHQ negatives had any syndromal mental illness. Depression was most common in all the groups and it was observed that substance abuse was higher among writers −29.4% (Table 4a and b).

**Table 4a:** General health questionnaire positivity among the groups

Variables	Controls	Musicians	Writers	F/χ^2^ value	*P* value
GHQ positive (cases)	13 (32.5)	14 (35%)	17 (42.5)	0.933	0.627
GHQ negative2 (non cases)	27 (67.5)	26 (65)	23 (57.5)		
creativity	6 (15)	40 (100)	33 (82.5)	71.65	0.001

Figures in parentheses are in percentage, GHQ – General health questionnaire

**Table 4b:** Scan diagnoses among groups

Variables	Controls (%)	Musicians (%)	Writers (%)
Depression#	11 (84.6)	11 (78.6)	10 (58.8)
Bipolar	Nil	Nil	Nil
Psychosis	Nil	1(7.1)	Nil
OCD	Nil	Nil	1 (5.9)
Adjustment	Nil	Nil	1 (5.9)
Somatoform	1 (7.7)	Nil	Nil
Substance dependence	1 (7.7)	2 (14.3)	5 (29.4)
Total	13 (100)	14 (100)	17 (100)

# Controls: major depression 5, dysthymia 6, Musicians; major depression 6, dysthymia 5, Writers: major depression 8, dysthymia 2

### Perceived stress

#### Coping patterns

On administering the coping check list which has 7 domains, namely problem focused, emotion focused, distraction, acceptance/ redefinition, religion/ faith, denial/ blame and social support, it was found that there was a significant difference between the groups in religion/faith domain [Table 6a].

The ten most common coping behaviors used by each group have been shown in the [Tables 6b, 6c, 6d]. It was noted that the coping skills no.s 1, 20,30,52,53 and 54 were common to all the three groups.

**Table 5:** Perceived stress score

Variable	Controls n=40	Musicians n=40	Writers n=40	F/χ^2^ value	*P* value
PSS total	23.05 ±4.44	20.1 ± 6.83	20.45 ± 7.73	2.46	0.089
PSS positive (=28)	6 (15%)	4 (10%)	9 (22.5%)	2.38	0.305
PSS negative (<28)	34 (85%)	36 (90%)	31 (77.5%)		

Stress profile across the groups was assessed using Cohen's perceived stress scale (PSS). Though writers had highest percentage of scores (22.5%) this did not reach statistical significance

**Table 6a:** Coping patterns

Variables	Controls n=40	Musicians n=40	Writers n=40	*F* value	*P* value
Problem focused	6.75 ± 1.9	6.2 ± 1.87	6.28 ± 1.68	1.08	0.34
Emotion focused	6.85 ± 2.94	6.58 ± 2.09	5.95 ± 2.70	1.23	0.29
Distraction	1.8 ± 1.57	1.63 ± 1.19	1.65 ± 1.14	0.44	0.65
Acceptance / redefinition	7.53 ± 1.82	7.70 ± 1.81	7.05 ± 1.92	1.32	0.27
Religion/faith	3.80 ± 2.2	3.45 ± 2.58	2.30 ± 2.31	4.40	.01#*
Denial/blame	3.80 ± 1.82	3.53 ± 2.3	3.53 ± 1.83	0.17	0.84
Social support	3.57 ± 1.64	3.0 ± 1.32	2.92 ± 1.27	2.52	0.09

*P*<0.05;

# Controls v/s writers,

* Musicians v/s writers,

$ Controls v/s musicians.

**Table 6b:** Ten most common coping skills adapted by controls

Item no.	Coping behavior	Frequency	Percentage
53	Analyze the problem and solve it bit by bit	39	97.5
54	Make a plan of action and follow it.	37	92.5
40	Help others in trouble or distress	35	87.5
1	You, go over the problem again and again in your mind, to try to understand it.	34	85
20	Try to look on the bright side of things.	34	85
27	Pray to God	34	85
30	Come up with a couple of different solutions to the problem.	34	85
45	Turn to work/studies to take your mind off things.	33	82.5
52	You know what has to be done so you double your efforts and try harder to make things work.	32	80
7	Seek reassurance and emotional support from family members.	32	80

**Table 6c:** Ten most common coping skills adapted by writers

Item no.	Coping behavior	Frequency	Percentage
20	Try to look on the bright side of things.	36	90
30	Come up with a couple of different solutions to the problem.	36	90
1	You, go over the problem again and again in your mind, to try to understand it	34	85
45	Turn to work/studies to take your mind off things.	32	80
53	Analyze the problem and solve it bit by bit	32	80
47	Find a purpose or meaning in your suffering.	31	77.5
52	You know what has to be done so you double your efforts and try harder to make things work.	31	77.5
18	Accept the next best thing to what you wanted.	31	77.5
54	Make a plan of action and follow it.	31	77.5
56	Draw on your past experience of similar situations.	30	75

**Table 6d:** Ten most common coping skills adapted by musicians

Item no.	Coping behavior	Frequency	Percentage
1	You, go over the problem again and again in your mind, to try to understand it.	34 (85%)	85
20	Try to look on the bright side of things	34	85
47	Find a purpose or meaning in your suffering	34	85
52	You know what has to be done so you double your efforts and try harder to make things work.	34	85
53	Analyze the problem and solve it bit by bit.	34	85
57	Take up or indulge in a hobby (music, art, etc.).	34	85
27	Pray to God	33	82.5
30	Come up with a couple of different solutions to the problem.	32	80
29	Listen to music for comfort.	31	77.5
28	Make light of the situation/refuse to get too serious about it	31	77.5

#### Personality profile

Personality characteristics were compared between all groups on NEOFFI. On neurotic dimension comparison it was found that musicians (*P*=0.011) and writer's (*P*=0.023) had high scores compared to noncreative population but there was no difference between the creative groups. Comparing the extroversion dimension most of the subjects in the creative population scored higher (n=37) (*P*<0.000) compared to the noncreative sample where scores were evenly distributed. On the Openness dimension, Agreeable dimension and Conscientiousness dimension similar results were established (*P*<0.01).

**Table 7a:** Personality profile

Variables	Controls mean ± SD	Musicians mean ± SD	Writers mean ± SD	F value *P* value
Neuroticism	27.23 ± 7.71	31.93 ± 7.68	31.30 ± 7.45	4.493	0.013#$
Extroversion	34.07 ± 7.26	45.88 ± 7.21	45.83 ± 6.82	36.667	0.000#$
Openness	28.25 ± 7.12	43.28 ± 6.08	45.25 ± 4.48	96.299	0.000#$
Agreeableness	30.23 ± 7.09	42.98 ± 7.65	43.50 ± 7.05	42.767	0.000#$
Conscientiousness	38.95 ± 6.11	45.83 ± 7.03	46.93 ± 6.36	17.616	0.000#$

*P*<0.05;

# Controls v/s writers,

* Musicians v/s writers,

$ Controls v/s musicians

**Table 7b:** Comparison across groups on neuroticism dimension

	Controls	Musicians	Writers	Groups	*P* value*	F value
Very low	-	-	-			
Low	2(5)	-	-	CV/SM	6.49	0.011
Average	10 (25)	6(15)	7(17.5)	CV/SW	5.143	0.023
High	17 (42.5)	12 (30)	12 (30)	WV/SM	0.088	0.766
Very high	11(27.5)	22 (55)	21 (52.5)			

C = Controls, W = Writers, M = Musicians.

* Manten Hanzel test linear association, Figures in parentheses are in percentage

**Table 7c:** Comparison across groups on extroversion dimension

	Controls	Musicians	Writers	Groups	*P* value*	F value
Very low	-	-	-			
Low	2(5)		1 (2.5)	CV/SM	27.179	0.012
Average	14(35)	2(5)	-	CV/SW	24.402	0.01
High	11 (27.5)	1 (2.5)	2 (5)	WV/SM	0.000	1.00
Very high	13 (32.5)	37 (92.5)	37 (92.5)			

C = Controls, W = Writers, M = Musicians.

* Manten Hanzel test linear association, Figures in parentheses are in percentage

**Table 7d:** Comparison across groups on openness dimension

	Controls	Musicians	Writers	Groups	*P* value*	F value
Very low	1 (2.5)	-				
Low	11(27.5)	-	-	CV/SM	40.072	0.001
Average	15 (37.5)	2(5)	-	CV/SW	45.083	0.001
High	6(15)	1 (2.5)	1 (2.5)	WV/SM	1.654	0.198
Very high	7(17.5)	37 (92.5)	39 (97.5)			

C = Controls, W = Writers, M = Musicians.

* Manten Hanzel test linear association, Figures in parentheses are in percentage

**Table 7e:** Comparison across groups on agreeableness dimension

	Controls	Musicians	Writers	Groups	*P* value*	F value
Very low	12 (30)	-	1 (2.5)			
Low	8(20)	4(10)	2(5)	CV/SM	30.004	0.001
Average	12 (30)	5 (12.5)	1 (2.5)-	CV/SW	34.725	0.001
High	4(10)	5 (12.5)	8(20)	WV/SM	0.610	0.435
Very high	4(10)	26 (65)	28 (70)			

C = Controls, W = Writers, M = Musicians.

* Manten Hanzel test linear association, Figures in parentheses are in percentage

**Table 7f:** Comparison across groups on conscientiousness dimension

	Controls	Musicians	Writers	Groups	*P* value*	F value
Very low	1 (2.5)	-	-			
Low	3 (7.5)	2 (5)	1 (2.5)	CV/SM	14.720	0.001
Average	12 (30)	3 (7.5)	2 (5)	CV/SW	23.057	0.001
High	17 (42.5)	8 (20)	5 (12.5)	WV/SM	1.339	0.247
Very high	7 (17.5)	27 (67.5)	32 (80)			

C = Controls, W = Writers, M = Musicians.

* Manten Hanzel test linear association, Figures in parentheses are in percentage

## DISCUSSION

The discussion aims at examining the various issues about creativity and mental health in relation to this study and in comparison with prior studies.

### Methodological issues

This study was a case control randomized study. The study sample were chosen from the directories of eminent writers and musicians after randomization and compared with a randomized sample from the general population. Assessments were done in the areas of mental illness, coping patterns and personality profile.

The sample size was 40 in each group, which is comparable to similar studies, done earlier, which have looked for mental illness in creative people.[9,10] Larger sample sized studies have been mostly retrospective biographical studies.[11]

The recruitment of subjects for the study was done from alphabetically organized directories from literature and music academies after randomization. This was unlike earlier studies most of which did not have randomized control sample.

All subjects chosen for the study willingly participated in the study. Nobody refused to give written informed consent.

### Sociodemographic findings

The sex distribution was equal across the groups, which eliminated any bias in terms of any domain assessed. The education level of the writers was mostly post graduation, which is similar to the findings of earlier studies.[11] The music group and the control group were comparable education wise.

In terms of birthplace and child hood stay, most of the writers were from rural background and musicians were from urban background as were the controls. Whether these had any advantages or disadvantages is not clear. The earlier studies have not looked at this data.

Most of the writers were married and were staying together so were control population but among the musicians staying single was more common which was in contrast to earlier studies.[1]

### Family characteristics

It was notable that 100% of musicians had history of creativity (music, dance, theater, literature) in the family. It was also significantly high in the writers. It was also seen that the children of these creative people pursued creative work either as a profession or as a hobby. Whether this suggests that creativity is a hereditary trait and hence runs in the families or there are environmental factors like being in creative surroundings getting more opportunities, including early exposure in the childhood and adequate training, is not clear. It is also worth mentioning that creativity in the spouses of creative population also was significantly higher than those of the control population.

Several authors have reported a high occurrence of mental illness among the relatives of the creative population.[9,11] In agreement with these studies we also found significantly higher rates of mental illness in the family especially mood disorders. In both groups of the creative population unlike these studies, which found higher rates of BPAD in 1st degree relatives, there was only one writer out of our whole creative population who had a family history of BPAD in our sample. However it should be noted that in the present study no structured tool was used to elicit family history of mental illness. It was based on the descriptive data given by the subjects, which may involve memory bias.

### Clinical correlates

The subjects were given GHQ-28 to screen for psychiatric caseness and then the GHQ positive cases were administered SCAN 2.1. The prevalence of mental illness across the groups was similar and was not found to be statistically significant. This finding was similar to earlier studies on creativity and psychopathology.[12]

Recent studies in 80's and 90's have shown artists and writers to have higher prevalence of mental illness.[9,11] It is to be noted that these studies, had studied biographies and none of them were randomized.

In our study we found that the majority of the people who were having mental illness suffered from depressive disorders (dysthymia, mild and moderate depression). This was in agreement with the earlier studies.[3,13–15] Some of the studies had found more of bipolar spectrum among creative people. We did not find this. Whether periods of hypomania were considered as bursts of inspiration, short lasting and enhancing creativity, hence subjects did not find anything abnormal in these periods needs to be looked in to.. However in our study when administered a structured interview (SCAN) none of them had any evidence of hypomania, cyclothymia either at present or in the past. Earlier studies found lesser prevalence of mental illness in creative people[16] and others found that it was comparable to general population.[10] In the present study one of the musicians was a diagnosed case of paranoid schizophrenia. He was in remission on treatment and was carrying on well with his profession. Similarly one of the writers had been diagnosed as having OCD and was on regular treatment. These findings were similar to recent Indian studies,[17] which found more of depressive disorders. Also in this study alcohol dependence was found to be more though not statistically significant in writers. Many writers have spoken about alcohol dependence being more in creative population.[15,18]

What is striking in the present study is that 7 out of 11 people(one with substance use, 6 with depressive disorders) in the control group had sought psychiatric help whereas except for 2 (one with OCD and one with schizophrenia) none of the experimental group had sought psychiatric help. There can be different explanations for this:

- The stigma of a well known person (writer or musician) contacting a psychiatrist would fetch them a ill-fame.
- The fear that taking neurotropics would hinder their creativity
- The popular notion that you have to be “little mad" to be creative
- The idea that the creative people are better equipped than the general population to cope with stressors (i.e., to handle their depression by themselves).

### Coping skills

There have been earlier studies,[10,19,20] which have spoken about effect of psychiatric treatment and illness on creativity. In one study by Schou[20] it was shown that treatment with Lithium hindered creativity in 12 persons, improved creativity in 6 and was unchanged in 6. It was also noted in Andreasen's study in 1987 that many writers reported that they were more creative in euthymic states rather than in an affective episode (which is again contrary to the popular beliefs).

In the present study, we studied the coping patterns in the 3 groups. To the best of our knowledge there are no major published studies regarding coping in creative people. In this study the musicians and controls were noticed to have more of religious and faith domain skills and differed significantly from writers. In musicians perhaps this can be explained by the basis of Indian Music (both Carnatic and Hindustani Music), which has a religious background. It was surprising that in spite of having music or writing as a skill/ talent most of them said they do not use it at the time of stress (As answers to items no. 50 and 57 appendix 5) and when they are free from stress only then they are able to produce creative work of good quality (Answer to item 50 appendix 5). This is again in contrast to the popular belief that writers and musicians come out with sudden ‘bursts of inspiration’ ‘madness or ‘intense emotions’ and then would create something great. Previous studies[9] have corroborated this finding. Andreasen's (1987) sample said they would prefer a euthymic mood state to write.[9] reported in his study that the 75% of writers told him that alcohol hindered their creativity.

Items 1, 20,30,52,53 and 54 were common to all the three groups. These belong to problem focused and acceptance redefinition domains.

### Stress profile

We studied the stress profile in the three groups by administering Cohen's perceived stress scale. Though earlier studies[21,22] have spoken about higher levels of stress and anxiety in creative population none of these studies have actually measured perceived stress using a structured instrument. In the present study the stress profile was comparable across the groups. Though writers had highest percentage of scores (22.5%) this did not reach statistical significance.

### Personality profile

In the present study the dimensional personality profile was assessed on NEO –FFI (1989) on the following domains. Neuroticism, extraversion, openness, agreeableness conscientiousness. It was notable that higher scores were found on each of these dimensions for the creative groups compared to non-creative group but there was no difference between the writers and the musicians. This was in agreement with[18] where she used international personality disorder examination scale (IPDE) and found no difference between artists and scientists regarding the personality profile.

The creative groups scored high on the openness dimension which was statistically significant which mean that the creative people were more aesthetic and differed significantly from the noncreative group in feelings, actions, ideas, values and fantasy. This is in agreement with the earlier studies.[21,23] On the dimension of agreeableness which consists of traits trust, straightforwardness, altruism, compliance, modesty, tender mindedness and Conscientiousness which comprises of competence order, dutifulness, achievement, self discipline, deliberation again musicians and writers scored high in agreement with the earlier studies.[24]

The questions that now arise are:

- Are these personality traits that make a person creative?
- Does the creativity make a person attain these traits
- Whether scoring high on these dimensions makes the creative groups more vulnerable to mental illness?
- Are they protected because of their creativity though they have higher scores on these dimensions?
- Is there no role of the personality characteristics in making a person more vulnerable to mental illness?

Across the studies,[4] it has been well established that highly creative individuals showed a characteristic pattern of interest which was interpreted as a concern for meaning and implies more cognitive flexibility, interest, accuracy in communication, intellectual curiosity and lack of interest in policing their own interests or those of others. The IPAR studies (1939) showed that highly creative individuals in different fields shared many personality characteristics.

Strengths of the study

- This was a randomized control study, which was conducted with personal interviews with the subjects.
- Structured instruments were used.
- Strict inclusion and exclusion criteria were used.
- An effort was made to study different aspects of creativity; l creativity and mental illness; stress profile and coping skills and two different creative groups were studied with a controlled population.
- The subjects who participated in the experimental group were accomplished writers and musicians many years of experience in their respective fields.

### Limitations

- The sample size was small and case to case matching was not done.
- Though 2 groups of creative people were studies the findings cannot be generalized to other creative groups.
- No scales to assess creativity were used.
- Family history was not assessed using structured instruments
- Local adaptation of NEOFFI and PSS was not done.

### Future directions

- Creativity and mental health needs to be studied in large epidemiological samples, which will overcome the ascertainment biases.
- Whether developing a hobby like dance, music or painting would be promoting mental health and better coping skills in general population needs to be looked into.
- There is a need to study other creative groups like theatre artists, painting artists, dancers and architects and to look into these aspects.
- It would be worthwhile studying the creative groups who take creative work as the sole profession and earn livelihood through it and studying whether they have different patterns of psychiatric morbidity and personality characteristics.
